# The effect of nirmatrelvir‐ritonavir on viral clearance and length of hospital stay in patients infected with SARS‐CoV‐2 omicron variants

**DOI:** 10.1111/irv.13095

**Published:** 2023-02-02

**Authors:** Yu Wang, Danyang Zhao, Xubo Chen, Xinbing Liu, Wenying Xiao, Liuliu Feng

**Affiliations:** ^1^ Department of Cardiology, Shidong Hospital, Yangpu District Shidong Hospital Affiliated to University of Shanghai for Science and Technology Shanghai China; ^2^ Department of Plastic and Reconstructive Surgery, Shanghai Ninth People's Hospital Shanghai Jiao Tong University School of Medicine Shanghai China; ^3^ Department of Rehabilitation medicine, Shidong Hospital, Yangpu District Shidong Hospital Affiliated to University of Shanghai for Science and Technology Shanghai China

**Keywords:** COVID‐19, nirmatrelvir‐ritonavir, omicron variant, Paxlovid, SARS‐CoV‐2

## Abstract

**Background:**

The pandemic of coronavirus disease 2019 (COVID‐19) has caused heavy burdens on national healthcare systems. Nirmatrelvir‐ritonavir (Paxlovid) may be one of the most promising therapeutic drugs, with reports of up to 89% reduction rates in hospitalization risk and death among patients with mild‐to‐moderate COVID‐19 who are at risk of developing severe disease. However, limited studies have investigated the effects of this class of drugs on viral clearance and length of hospital stay.

**Methods:**

In this study, we retrospectively analyzed the characteristics of patients infected with the Omicron variant of severe acute respiratory syndrome coronavirus 2 (SARS‐CoV‐2) and investigated the effects of oral nirmatrelvir‐ritonavir on viral clearance and length of hospital stay in mild‐to‐moderate COVID‐19 patients at high risk for progression to severe disease.

**Results:**

The median SARS‐CoV‐2 negative conversion time was 16 (13–20) versus 13 (10–16) days (control group versus nirmatrelvir‐ritonavir group, *p* < 0.001), the median length of hospital stay was 13 (10–16) versus 12 (13–14) days (control group versus nirmatrelvir‐ritonavir group, *p* = 0.01), and the SARS‐CoV‐2 negative conversion time and length of hospital stay were significantly shorter in the nirmatrelvir‐ritonavir group than in the control group. When controlling for hypertension, chronic kidney disease, severity status of COVID‐19, use of antibiotic agent, and COVID‐19 vaccine received, multiple stepwise linear regression analysis showed that nirmatrelvir‐ritonavir treatment was negatively associated with the SARS‐CoV‐2 negative conversion time and length of hospital stay.

**Conclusion:**

Nirmatrelvir‐ritonavir reduces the viral clearance time and length of hospital stay in hospitalized patients with COVID‐19. Nirmatrelvir‐ritonavir might be a promising drug to reduce the virus load and the heavy burden of healthcare systems.

## 1 INTRODUCTION

The pandemic of coronavirus disease 2019 (COVID‐19) has caused heavy burdens on national healthcare systems. With increasing morbidity and death counts, the need for novel and effective oral antiviral drugs for the control of COVID‐19 is unmet. Nirmatrelvir‐ritonavir (Paxlovid) may be one of the most promising therapeutic drugs, with reports of up to 89% reduction rates in hospitalization risk and death among patients with mild‐to‐moderate COVID‐19 who are at risk of developing severe disease.^1^ As a combination protease inhibitor, nirmatrelvir‐ritonavir targets the major protease of severe acute respiratory syndrome coronavirus 2 (SARS‐CoV‐2) to block viral replication.^2^, ^3^ However, limited clinical studies have evaluated the effects of nirmatrelvir‐ritonavir use in patients infected with SARS‐CoV‐2 omicron variants, and studies focusing on the drugs' effects on virus clearance are also limited.

## 2 METHOD

In this retrospective observational study, we investigated the effects of oral nirmatrelvir‐ritonavir on viral clearance and length of hospital stay in mild‐to‐moderate COVID‐19 patients at high risk for progression to severe disease. All data were obtained from the medical records of patients infected with the SARS‐CoV‐2 Omicron variant between April 12, 2022 and June 15, 2022 in Shidong hospital. In total, 760 patients were included. SARS‐CoV‐2 infection was diagnosed via real‐time polymerase chain reaction (RT‐PCR) from nasopharyngeal swabs. Patients who have at least one characteristic or condition associated with high risk of progression to severe Covid‐19 were included.^1^ The exclusion criteria were as follows: severe or critical COVID‐19, transfer to another hospital, lack of data regarding the duration of virus clearance, and/or length of hospital stay, and missing data. The study was approved by the Medical Ethics Committee of Shidong Hospital. The requirement for individual consent for this retrospective study was waived.

COVID‐19 severity status was classified as mild and moderate in accordance with the Diagnosis and Treatment Scheme for COVID‐19. Mild COVID‐19 was characterized by fever and respiratory symptoms with no radiological evidence of pneumonia, while moderate COVID‐19 was characterized by fever and respiratory symptoms with radiological evidence of pneumonia. Patients who were treated with nirmatrelvir‐ritonavir were administered every 12 h for 5 days. The study outcomes included nucleic acid test negative conversion time and length of hospital stay. Negative conversion was defined as both negative RT‐PCR results (cycle threshold value ≥35 for ORF1ab and N genes) on two consecutive days. The criteria for discharge from the hospital were negative conversion of nucleic acid test and disease improvement.

Data were shown as numerical values, mean ± standard deviation, or median (interquartile range). Continuous variables were analyzed using the Mann–Whitney *U* test or a *t* test. Categorical variables were analyzed using the chi‐square test to compare differences between the two groups. Kaplan–Meier curve was used to depict the negative conversion time of viral RNA, and log‐rank test was used to compare the conversion time between the no nirmatrelvir‐ritonavir and nirmatrelvir‐ritonavir groups. The coefficient and 95% confidence interval (CI) for each significant variable were determined using multiple linear regression analysis. A two‐sided *P*‐value of <0.05 between the two groups was considered statistically significant. Statistical analyses were performed using the IBM SPSS software (version 25.0).

## 3 RESULTS

**FIGURE 1:**
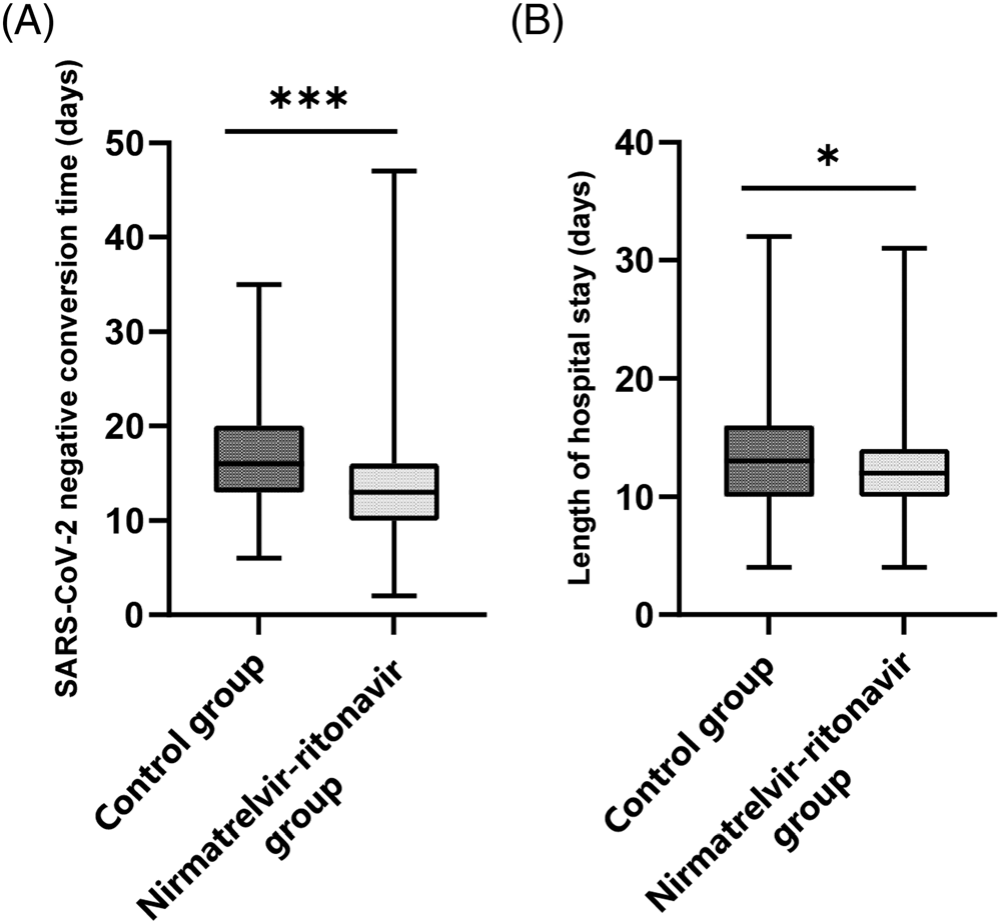
The severe acute respiratory syndrome coronavirus 2 (SARS‐CoV‐2) negative conversion time and length of hospital stay of the nirmatrelvir‐ritonavir group and control group. *** *P* < 0.001, **P < 0.01, *P < 0.05

**FIGURE 2:**
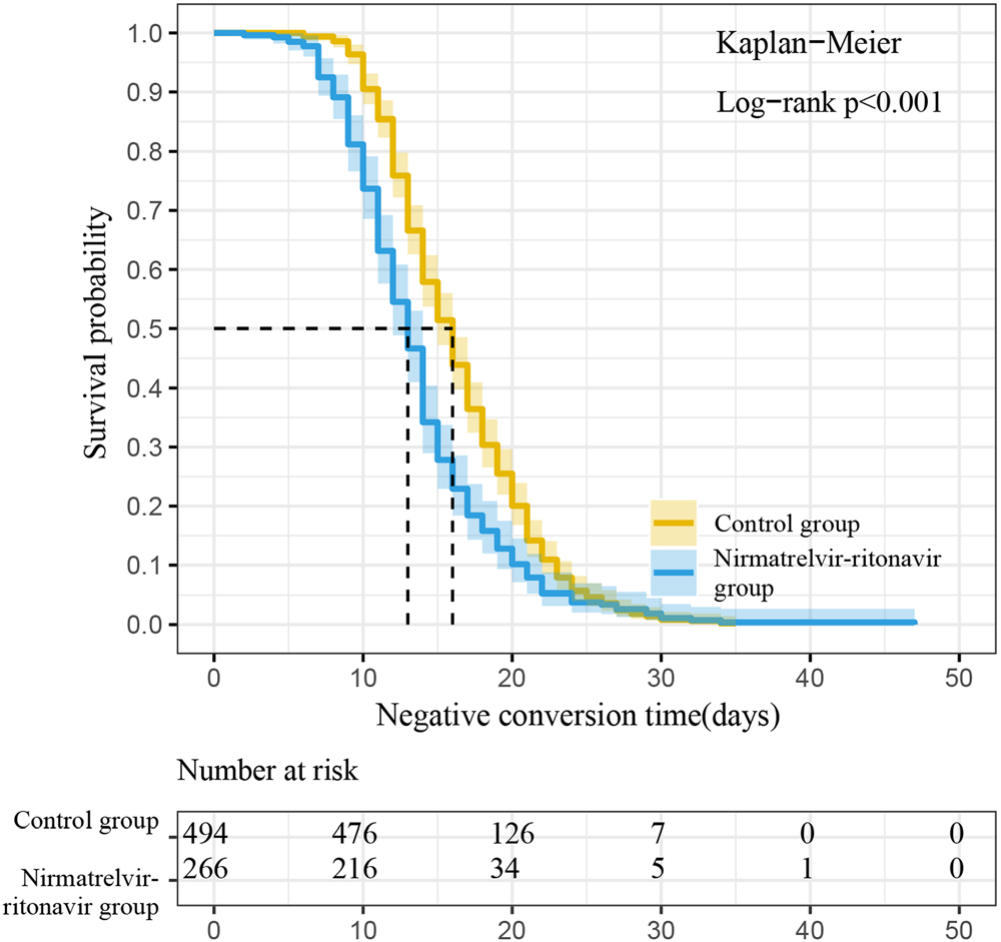
Kaplan–Meier curves showing time to negative conversion of viral RNA

The characteristics of patients were shown in Table 1; of the 760 patients, 266 received Paxlovid (nirmatrelvir‐ritonavir group) and 494 received no nirmatrelvir‐ritonavir (control group). The mean age of our study patients (332 male and 428 female patients) was 76.37 ± 12.49 years. As presented in Table 1, there were no significant differences in age, sex, symptoms before treatment (fever, cough, sputum, myalgia, sore throat, headache, dyspnea, and diarrhea), treatment with glucocorticoids, or coexisting diseases (diabetes, coronary artery disease, cerebrovascular disease, and chronic obstructive pulmonary disease) between the nirmatrelvir‐ritonavir and control groups. The differences in the SARS‐CoV‐2 negative conversion time, length of hospital stay, hypertension, chronic kidney disease, severity status of COVID‐19, use of antibiotic agent, and COVID‐19 vaccine received between the two groups were statistically significant. The median SARS‐CoV‐2 negative conversion time was 16 (13–20) versus 13 (10–16) days (control group versus nirmatrelvir‐ritonavir group, *p* < 0.001); the median length of hospital stay was 13 (10–16) versus 12 (10–14) days (control group versus nirmatrelvir‐ritonavir group, *p* = 0.01) (Figure 1). The SARS‐CoV‐2 negative conversion time and length of hospital stay were significantly shorter in the nirmatrelvir‐ritonavir group than in the control group. Figure 2 shows that SARS‐CoV‐2 negative conversion time was significantly shorter in the nirmatrelvir‐ritonavir group than in the control group (log‐rank, *p* < 0.001).

**TABLE 1:** Characteristics of patients

Variables	Control group (*n* = 494, 65%)	Nirmatrelvir‐ritonavir group (*n* = 266, 35%)	*P*‐value
Age (years)	77 (67–86)	80 (69–88)	0.197
Duration of viral RNA‐negative conversion (days)	16 (13–20)	13 (10–16)	<0.001
Hospitalization (days)	13 (10–16)	12 (10–14)	0.010
Sex			0.070
Female	290 (58.7%)	138 (51.9%)	
Male	204 (41.3%)	128 (48.1%)	
COVID‐19 vaccine received	70 (14.2%)	67 (25.2%)	<0.001
Hypertension	262 (53%)	162 (60.9%)	0.037
Diabetes	120 (24.3%)	59 (22.2%)	0.513
Coronary artery disease	101 (20.4%)	58 (21.8%)	0.660
Cerebrovascular disease	89 (18%)	62 (23.3%)	0.081
Chronic obstructive pulmonary disease	47 (9.5%)	18 (6.8%)	0.196
Chronic kidney disease	37 (7.5%)	8 (3%)	0.013
Severity status of COVID‐19			<0.001
Mild	331 (67%)	134 (50.4%)	
Moderate	163 (33%)	132 (49.6%)	
Fever	97 (19.6%)	47 (17.7%)	0.509
Cough	353 (71.5%)	189 (71.1%)	0.906
Sputum	281 (56.9%)	150 (56.4%)	0.896
Myalgia	32 (6.5%)	11 (4.1%)	0.182
Sore throat	74 (15%)	51 (19.2%)	0.137
Headache	6 (1.2%)	3 (1.1%)	1.000
Dyspnea	31 (6.3%)	20 (7.5%)	0.513
Diarrhea	6 (1.2%)	3 (1.1%)	1.000
Use of antibiotic agent	134 (27.1%)	54 (20.3%)	0.038
Treatment with glucocorticoid	12 (2.4%)	12 (4.5%)	0.117

*Note*: Data are presented as number (%) or median (interquartile range). Continuous variables use Mann–Whitney *U* test, and categorical variables use chi‐squared test for comparing the characteristics of nirmatrelvir‐ritonavir and control groups.

Abbreviation: COVID‐19, coronavirus disease 2019.

In addition, multiple stepwise linear regression analysis showed that when controlling for hypertension, chronic kidney disease, severity status of COVID‐19, use of antibiotic agent, and COVID‐19 vaccine received, nirmatrelvir‐ritonavir treatment was negatively associated with the SARS‐CoV‐2 negative conversion time (coefficient, −2.225; 95% CI: −2.996 to −1.455; *P <* 0.001) and length of hospital stay (coefficient, −0.784; 95% CI: −1.542 to −0.027; *P =* 0.043) (Table 2). This also showed that hospitalized patients with COVID‐19 who received nirmatrelvir‐ritonavir had significantly more rapid viral clearance and shorter length of hospital stay than those who did not receive nirmatrelvir‐ritonavir.

**TABLE 2:** Association between two outcomes with nirmatrelvir‐ritonavir treatment

Variable	SARS‐CoV‐2 negative conversion time				Length of hospital stay			
	Coefficient	95% CI (lower‐upper)		*P*‐value	Coefficient	95% CI (lower‐upper)		*P*‐value
COVID‐19 vaccine received	−0.429	−0.805	−0.052	0.026	−0.263	−0.633	0.108	0.164
Hypertension	−0.220	−0.940	0.501	0.550	−0.671	−1.379	0.038	0.063
Severity status of COVID‐19	0.578	−0.179	1.336	0.134	−0.461	−1.206	0.284	0.225
Chronic kidney disease	2.506	0.990	4.021	0.001	0.945	−0.545	2.435	0.213
Use of antibiotic agent	1.937	1.091	2.784	<0.001	1.466	0.633	2.299	0.001
Nirmatrelvir‐ritonavir treatment	−2.225	−2.996	−1.455	<0.001	−0.784	−1.542	−0.027	0.043

Abbreviations: CI, confidence interval; COVID‐19, coronavirus disease 2019; SARS‐CoV‐2, severe acute respiratory syndrome coronavirus 2.

## 4 DISCUSSION

Nirmatrelvir‐ritonavir is an important treatment that can be added to therapeutic regimens for early treatment of COVID‐19 to reduce incidence of severe disease, hospitalization, and/or death.^4^, ^5^ A previous study in healthy adults showed that nirmatrelvir was well tolerated, safe, and effective in increasing plasma concentrations.^6^ Fangfang Sun et al. reported that early administration of nirmatrelvir‐ritonavir within 5 days since diagnosis is associated with a faster clearance of viral load and a shorter time to viral elimination compared with administration of nirmatrelvir‐ritonavir beyond 5 days in high‐risk COVID‐19 patients who are immunocompromised.^7^ Further, early initiation of nirmatrelvir‐ritonavir was associated with reduced risks of mortality, risk of hospitalization, and in‐hospital disease progression.^8^ However, limited published clinical studies exploring the effects of nirmatrelvir‐ritonavir treatment on SARS‐CoV‐2 nucleic acid negative conversion time and length of hospital stay. Recently, a cohort study indicated that early treatment with nirmatrelvir‐ritonavir could significantly reduce risks of COVID‐19 development, progression, and all‐cause mortality, and nirmatrelvir‐ritonavir‐treated hospitalized patients showed faster decreased viral loads than those not treated with nirmatrelvir‐ritonavir.^9^ Our study showed that nirmatrelvir‐ritonavir was associated with decreased SARS‐CoV‐2 negative conversion time. Moreover, we found that nirmatrelvir‐ritonavir use could also reduce the length of hospital stay. These results support the use of nirmatrelvir‐ritonavir for hospitalized COVID‐19 patients with SARS‐CoV‐2 Omicron variant. Due to that viral shedding persists for a longer period will prolong deisolation and hospital stay thus increase the financial burden, nirmatrelvir‐ritonavir might be a promising drug to reduce the virus load and the heavy burden of healthcare systems.

Previous studies suggested that SARS‐CoV‐2 Omicron variant caused very high risk of infection but less severe cases or death^10^, ^11^; in this study, there were no hospitalized patients who died or experienced severe COVID‐19, so we did not analyze the effect of nirmatrelvir‐ritonavir treatment on reducing the progression of severe COVID‐19 and mortality. Case reports have documented that some patients who have completed one course (5 days) of nirmatrelvir‐ritonavir experienced rebound of COVID‐19 infections^12^, ^13^, ^14^; recently, we also reported a rapid COVID‐19 rebound in a severe COVID‐19 patient during a 20‐day course of nirmatrelvir‐ritonavir.^15^ This study was unable to analyze the association between nirmatrelvir‐ritonavir use and recurrence of COVID‐19 symptoms because a follow‐up retrospective study was lacking. Moreover, another limitation was that data on viral load were lacking in our study; therefore, the effect of nirmatrelvir‐ritonavir use on viral load could not be analyzed.

## 5 CONCLUSION

Nirmatrelvir‐ritonavir reduces the viral clearance time and length of hospital stay in hospitalized patients with COVID‐19. Nirmatrelvir‐ritonavir might be a promising drug to reduce the virus load and the heavy burden of healthcare systems.

## AUTHOR CONTRIBUTIONS

**Yu Wang:** Conceptualization; data curation; formal analysis; investigation; methodology. **Danyang Zhao:** Conceptualization; data curation; formal analysis; investigation; methodology. **Xubo Chen:** Conceptualization; data curation; formal analysis; investigation; methodology. **Xinbing Liu:** Conceptualization; data curation; investigation; methodology. **Wenying Xiao:** Conceptualization; data curation; investigation; methodology. **Liuliu Feng:** Conceptualization; data curation; investigation; methodology.

## CONFLICT OF INTEREST

None.

### PEER REVIEW

The peer review history for this article is available at https://publons.com/publon/10.1111/irv.13095.

## Data Availability

The datasets generated and/or analyzed during the current study are available from the corresponding author on reasonable request.
